# The impact of immunotherapy on the prognosis of small cell carcinoma of the esophagus: a propensity score-matched analysis of a retrospective study

**DOI:** 10.3389/fimmu.2025.1634834

**Published:** 2025-08-22

**Authors:** Bing Wang, Tianxing Liu, Zhe Yang, Hui Zhu, Fengge Zhou, Li Li, Bin Feng, Wang Jing

**Affiliations:** ^1^ Department of Medical Oncology, Shandong Provincial Hospital Affiliated to Shandong First Medical University, Jinan, China; ^2^ Shandong University, Shandong Provincial Hospital, Jinan, China; ^3^ Department of Radiation Oncology, Shandong Provincial Hospital Affiliated to Shandong First Medical University, Jinan, China; ^4^ Department of Radiation Oncology, Shandong Cancer Hospital & Institute, Shandong First Medical University & Shandong Academy of Medical Sciences, Jinan, China; ^5^ Tumor Research and Therapy Center, Shandong Provincial Hospital affiliated to Shandong First Medical University, Jinan, China; ^6^ Department of Oncology, Affiliated Hospital of Southwest Medical University, Luzhou, China; ^7^ Southwest Medical University, Luzhou, China; ^8^ Department of Radiation Oncology, Affiliated Hospital of Shandong University of Chinese Medicine, Jinan, China

**Keywords:** immunotherapy, small cell carcinoma of the esophagus, propensity score matching, prognosis, overall survival, progression free survival

## Abstract

**Objective:**

Small cell carcinoma of the esophagus (SCCE) is a rare neuroendocrine malignancy with no standardized treatment regimen. This study aims to evaluate the impact of immunotherapy on the prognostic survival outcomes of patients with SCCE.

**Methods:**

A retrospective analysis was conducted on 83 SCCE patients treated at the Provincial Hospital of Shandong First Medical University and the Cancer Hospital of Shandong First Medical University from January 2020 to June 2024. Propensity score matching (PSM) was applied to minimize potential biases between patients who received combination immunotherapy and those who did not. Survival outcomes, including overall survival (OS) and progression-free survival (PFS), were assessed using the Kaplan-Meier method, while univariate and multivariate analyses were performed using the Cox proportional hazards model.

**Results:**

Among the 83 patients included, 33 received combination immunotherapy and 50 did not. Prior to PSM, clinicopathological comparisons revealed that tumor size was significantly larger in the non-immunotherapy group (P = 0.032), and the immunotherapy group had more advanced N stages (P = 0.015). After 1:1 PSM, 20 matched pairs were analyzed. The immunotherapy group demonstrated a significantly longer OS compared to the non-immunotherapy group (22 months *vs*. 13 months, P = 0.0165), though PFS differences were not statistically significant (9 months *vs*. 7 months, P > 0.05). Univariate and multivariate analyses identified treatment methods (P=0.039) as independent prognostic factors for OS. Survival rate analysis showed that patients in the immunotherapy group achieved superior six-month (97.0% *vs*. 83.7%), one-year (78.9% *vs*. 56.5%), and two-year (22.1% *vs*. 10.4%) survival rates compared to the non-immunotherapy group.

**Conclusions:**

This study demonstrates that combination immunotherapy significantly improves overall survival in SCCE patients and represents an effective treatment strategy for this rare malignancy.

## 1 Introduction

Esophageal cancer is one of the most prevalent gastrointestinal malignancies worldwide, with three main pathological types: squamous cell carcinoma, adenocarcinoma, and small cell carcinoma of the esophagus (SCCE). As a high-grade neuroendocrine malignancy, SCCE demonstrates aggressive biological behavior with dismal prognosis, representing merely 0.5%-2.8% of all esophageal cancers. Current survival statistics reveal 5-year survival rates approximating 10% for limited-stage disease and approaching zero in extensive-stage presentations (1–3). Notably, epidemiological surveillance indicates an upward incidence trend of SCCE in contemporary cohorts, though this remains under characterized in population-based studies (4, 5). The evidence base for SCCE management suffers from critical knowledge gaps, particularly regarding prospective clinical trial data and consensus treatment guidelines (5). Current therapeutic paradigms are empirically derived from protocols established for: 1) conventional esophageal carcinomas, and 2) small cell lung cancer (sharing neuroendocrine lineage) (6). Platinum-based doublet chemotherapy forms the cornerstone of systemic treatment, typically involving Etoposide or Irinotecan in combination with Cisplatin (7). Multimodal approaches incorporating thoracic radiotherapy demonstrate particular efficacy in achieving locoregional control, with emerging data supporting concurrent chemoradiation for limited-stage disease (8). In the treatment of esophageal cancer, chemotherapy and radiotherapy remain the mainstays of treatment. However, with evolving oncology paradigms, immunotherapy has emerged as a promising fourth modality alongside surgery, chemotherapy, and radiotherapy. Immune checkpoint inhibitors (ICIs), such as Pembrolizumab and Nivolumab, have demonstrated survival benefits in various malignancies and are increasingly incorporated into perioperative oncology treatment strategies (8). Although these immunotherapeutic agents hold potential for SCCE, large-scale clinical studies specific to SCCE patients are lacking. Early studies suggest that combining immunotherapy with other therapeutic modalities may offer improved outcomes for SCCE patients (9). The objective of this study was to systematically analyze and summarize the data of SCCE (Jan 2019-June 2024) from the Provincial Hospital of Shandong First Medical University and the Tumor Hospital of Shandong First Medical University, through retrospective analyses, PSM, survival analyses, univariate and multivariate regression analysis, with the aim of offering novel insights and further substantiating the question of whether the combination of immunotherapy and other therapeutic modalities can enhance the prognosis of patients diagnosed with SCCE.

## 2 Materials and methods

### 2.1 Patient selection

**Figure 1:**
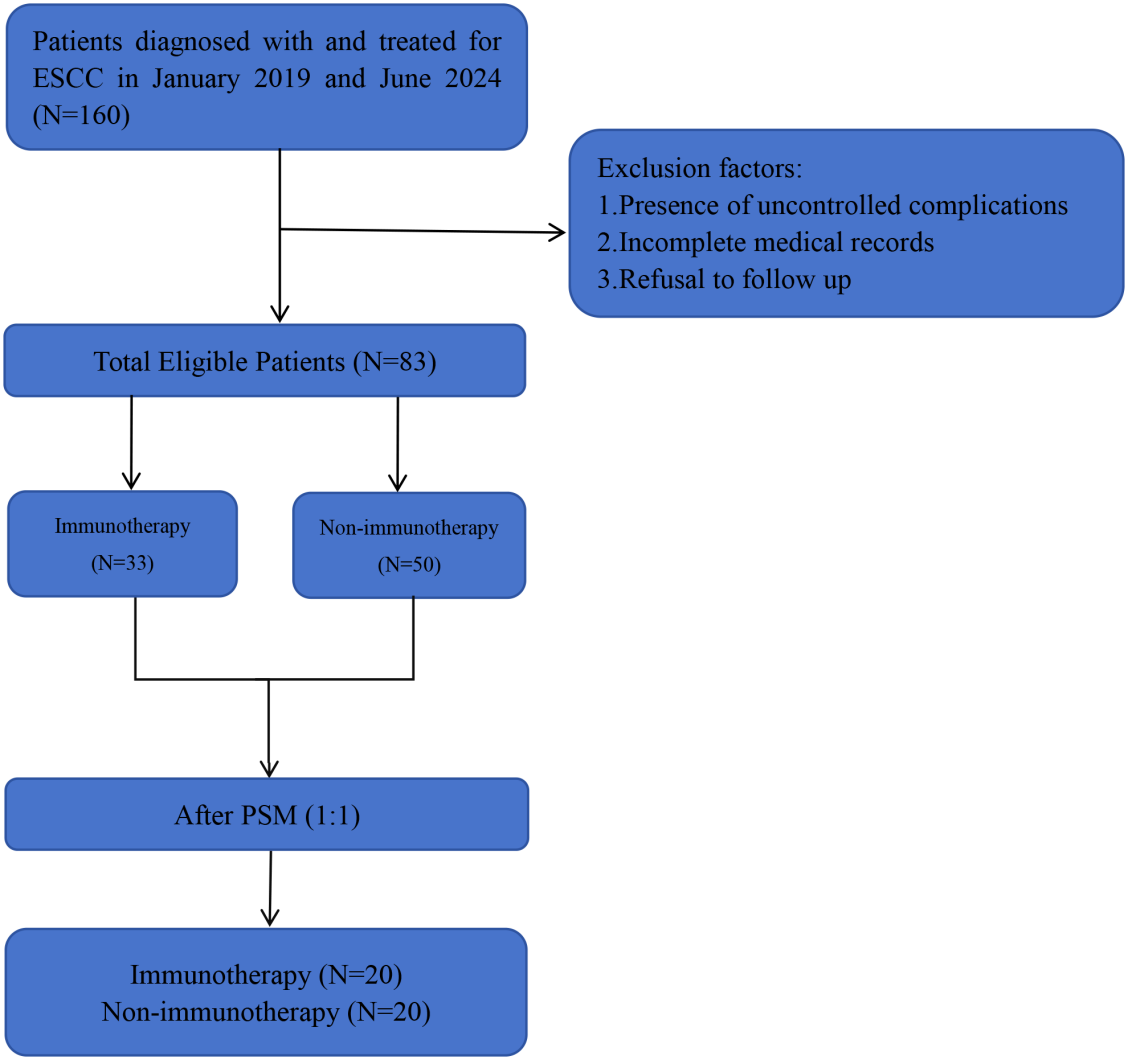
The study flowchart. SCCE, small cell carcinoma of the esophagus. PSM, propensity score matching analyses. Immunotherapy, other treatment modalities combined with immunotherapy (the same as below). Non-immunotherapy, no combination immunotherapy (the same as below).

This retrospective analysis included 160 histologically confirmed small cell carcinoma of the esophagus (SCCE) cases diagnosed between January 2019 and June 2024 at two tertiary institutions affiliated with Shandong First Medical University: the Provincial Hospital (n=25, 15.6%) and the Cancer Hospital (n=135, 84.4%). Of the 160 initially identified cases, 25 originated from Provincial Hospital and 135 from Cancer Hospital. **Figure 1** presents the comprehensive patient selection flowchart. Following rigorous application of inclusion/exclusion criteria, 83 patients (51.8% of initial cohort) were included in the final cohort, stratified into immunotherapy-administered (n=33) and non-immunotherapy (n=50) groups. Notably, 90.4% (75/83) of the study population presented with advanced-stage disease (stage III/IV) at diagnosis. Patient inclusion criteria (1) SCCE confirmed by histopathology (2) complete medical records (3) patient and family cooperation in follow-up. Patient exclusion criteria: (1) Presence of uncontrolled comorbidities (e.g., psychiatric, metabolic) (2) Incomplete medical records (3) Refusal of follow-up by the patient and family. The American Joint Committee on Cancer (AJCC) 8th edition TNM staging system was used in this study. This study was conducted in accordance with the Declaration of Helsinki.

### 2.2 Treatment groups

In this study, patients who never used immunotherapy drugs from the beginning of treatment to the end of treatment(regardless of the reason for the end of treatment)were defined as the “Non-immunotherapy group”.Conversely, patients were classified as the “Immunotherapy group” if they received at least two cycles of immunotherapy from the beginning to the end of their treatment. Among the 83 patients,50 were in the Non-immunotherapy group, while 33 were in the Immunotherapy group. Of the 33 patients in the Immunotherapy group, 13 received first-line combination immunotherapy, while the remaining 20 were treated with second-, third-, and fourth-line immunotherapy (12 second-line, 7 third-line, and 1 fourth-line).

Among the 50 patients in non-immunotherapy group, the first-line treatment regimen choice of single-agent Etoposide or Etoposide (E) in combination with Cisplatin (P) or Carboplatin (C) was 78% of the patients, which included simultaneous or sequential radiotherapy with EP/EC, or chemotherapy with EP/EC in combination with surgery, while first-line treatment regimens for the remaining 22% of the patients included surgery, radiotherapy alone, interventional embolization chemotherapy, Fluorouracil or Docetaxel in combination with Cisplatin. The immunotherapy drugs applied to the 33 patients with combination immunotherapy mainly included: Camrelizumab (24.2%), Serplulimab (21.2%), Toripalimab (12.1%), Sintilimab (12.1%), Envafolimab (6.0%), Pembrolizumab (6.0%), Tislelizumab (6.0%), Adebrelimab (3.0%). Of these 33 patients, 31 (93.9%) were treated with chemotherapy in combination with immunotherapy, and 11 (33.3%) were treated with chemotherapy and synchronous or sequential radiotherapy in combination with immunotherapy. Of these 31 patients, 12 (36.4%) chose Etoposide (E), platinum-based combination immunotherapy, 12 (36.4%) chose Albumin-bound paclitaxel, platinum-based combination immunotherapy, 5 (15.2%) chose Iriontecan, platinum-based combination immunotherapy, and 2(6.1%) patients respectively were treated with Docetaxel, platinum-based combination immunotherapy, and single agent capecitabine combination immunotherapy. In addition to the 31 patients mentioned above, 2 (6.1%) chose targeted combination immunotherapy, Anlotinib and Surufatinib. (Platinum-based chemotherapy mentioned in this study includes Cisplatin, Carboplatin, Nedaplatin, Lobaplatin).

### 2.3 Follow-up

All 83 patients with small cell carcinoma of the esophagus (SCCE) underwent systematic post-treatment surveillance through clinic visits and telephone-based follow-up assessments. Disease monitoring incorporated a standardized protocol including: physical examinations, contrast-enhanced or non-contrast computed tomography (CT) of the neck and thorax, ultrasonography for cervical/supraclavicular lymph node evaluation when clinically indicated, barium esophagography, 18F-FDG PET-CT, esophagogastroduodenoscopy, whole-body magnetic resonance imaging (WB-MRI), and technetium-99m bone scintigraphy. The final follow-up date for survival analysis was November 1, 2024.

### 2.4 Study endpoints

The primary endpoints of this study consisted of overall survival (OS) and progression-free survival (PFS) in 83 patients. OS was calculated as the time from the date of diagnosis to the date of last follow-up or death from any cause, whichever occurred first. PFS in the non-immunotherapy group was calculated as the time from treatment initiation to disease progression or death from any cause, whichever occurred first. PFS in the immunotherapy group was calculated as the time from first immunotherapy dose to disease progression or death from any cause, whichever came first.

### 2.5 Statistical analysis

Continuous variable data are described as means ± standard deviation while categorical variables are described as numbers (percentages). Differences in clinicopathological characteristics between the two groups were analyzed using t-tests for continuous variables and Pearson’s χ² test for categorical variables. To mitigate selection bias and confounding effects between the combination immunotherapy cohort and control cohort, propensity score matching (PSM) was implemented using a nearest-neighbor algorithm (1:1 ratio, caliper width=0.2). The propensity score model incorporated clinically relevant covariates: age at diagnosis, gender, body mass index (BMI), marital status (as a surrogate for social support), tumor characteristics (location, maximum diameter, TNM-8 staging), histologic differentiation grade, and comorbid conditions including hypertension, diabetes mellitus, chronic obstructive pulmonary disease, pneumonia, cerebral vascular accidents, and coronary artery disease. Survival outcomes were analyzed through Kaplan-Meier methodology with intergroup comparisons performed via log-rank testing. Prognostic determinants for overall survival (OS) and progression-free survival (PFS) were identified through univariable and multivariable Cox proportional hazards regression models. P-values less than 0.05 were considered statistically significant. Univariate and multivariate Cox regression analyses were used to identify independent prognostic factors for OS and PFS in the SCCE cohort, wherein variables exhibiting p-values less than 0.2 in univariable analysis were included in the multivariable analysis. All statistical analyses were performed using GraphPad Prism 8.3.0 and SPSS 26.0.

## 3 Results

A total of 83 patients with SCCE were included in this study, with a median age of 67 years, more than 90% of whom were stage III and IV patients. Of the cohort 16 patients(19.3%) were female, 67 patients(80.7%) were male, and the percentage of patients with comorbidities such as hypertension, diabetes mellitus, coronary artery disease, arrhythmia, and pleural effusion was 52 patients (62.7%), and the percentage of patients without comorbidities was 31 patients (37.3%).

### 3.1 Baseline characteristics of patients before PSM

**Figure 2:**
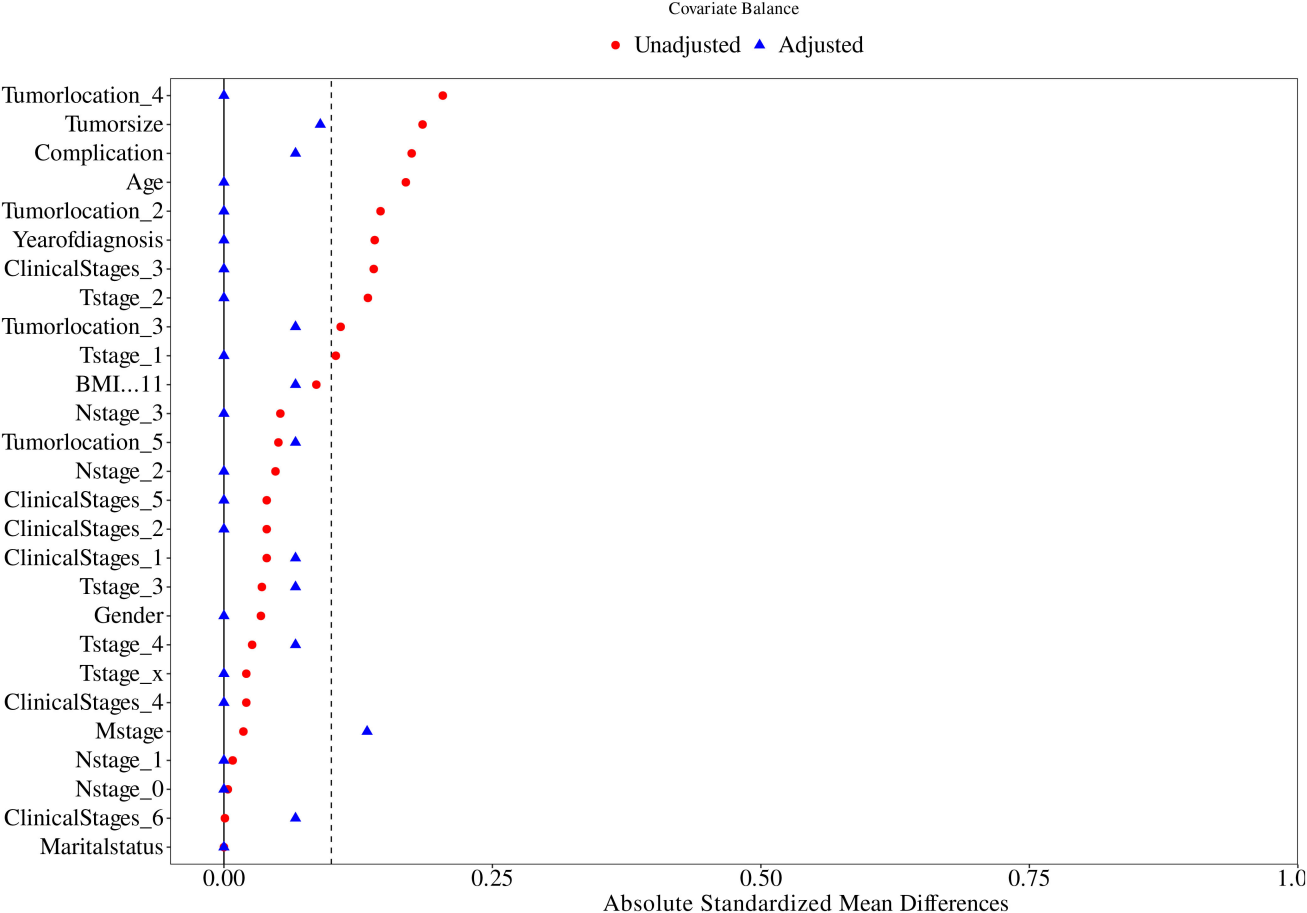
Propensity score matching between the Immunotherapy group and Non-immunotherapy group described using standardized mean difference (SMD).

As shown by the results of the comparison of the clinicopathological characteristics of patients before PSM (**Table 1**), at the 5% significance level, the differences were not statistically significant (P > 0.05) when comparing the Immunotherapy group with the Non-immunotherapy group in terms of age, BMI, gender, marital status, clinical stages, T stage, M stage, complication, and tumor location. Patients in the Non-immunotherapy group had larger tumors than those in the Immunotherapy group (t = 4.793, P = 0.032); patients in the Immunotherapy group had advanced N stage than those in the Non-immunotherapy group (x^2^ = 10.420, P = 0.015).This study used PSM to minimize the imbalance confounders between the two groups. The matching quality was assessed using standardized mean difference (SMD) (10).Covariates with SMD < 0.25 are considered moderately balanced (11), and those with SMD < 0.1 are considered highly balanced (12).The SMD between the matched cohorts were below 0.1, while below 0.25 in the M stage, as illustrated in **Figure 2**.

**Table 1:** Baseline characteristics before PSM.

Characteristics		Total	Immunotherapy (n=33)	Non-immunotherapy (n=50) _(1)_	P-value
Tumor size (cm)		5.42 ± 3.42	4.33 ± 1.65	6.19 ± 4.09	0.032
Age		66.16 ± 8.42	64.33 ± 8.45	67.36 ± 8.26	0.109
BMI		21.80 ± 3.60	22.47 ± 3.28	21.35 ± 3.77	0.170
Gender	Male	67 (80.72)	25 (75.76)	42 (84.00)	0.352
	Female	16 (19.28)	8 (24.24)	8 (16.00)	
Marital status	Single	2 (2.41)	1 (3.03)	1 (2.00)	0.765
	Married	81 (97.59)	32 (96.97)	49 (98.00)	
Clinical Stages	II	2 (2.53)	1 (3.33)	1 (2.17)	0.932
	IIIA	3 (3.80)	1 (3.33)	2 (4.35)	
	IIIB	20 (25.32)	7 (23.33)	13 (28.26)	
	IVA	4 (5.06)	1 (3.33)	3 (6.52)	
	IVB	47 (59.49)	20 (66.67)	27 (58.70)	
T stage	T0-1	5 (10.64)	0 (0.00)	5 (10.64)	0.323
	T2	13 (27.66)	5 (16.67)	8 (17.02)	
	T3	45 (95.74)	19 (63.33)	26 (55.32)	
	T4	14 (29.79)	6 (20.00)	8 (17.02)	
N stage	N0	5 (6.49)	2 (6.67)	3 (6.38)	0.015
	N1	31 (40.26)	6 (20.00)	25 (53.19)	
	N2	26 (33.77)	12 (40.00)	14 (29.79)	
	N3	15 (19.48)	10 (33.33)	5 (10.64)	
M stage	M0	30 (35.29)	11 (36.67)	19 (39.58)	0.797
	M1	48 (48.48)	19 (63.33)	29 (60.42)	
Complication	Yes	51 (51.52)	22 (66.67)	29 (58.00)	0.427
	No	32 (94.12)	11 (33.33)	21 (42.00)	
Tumor location	1	2 (5.88)	1 (3.03)	1 (2.04)	0.682
	2	6 (6.32)	1 (3.03)	5 (20.20)	
	3	34 (35.79)	15 (45.45)	19 (38.78)	
	4	17 (17.89)	8 (24.24)	9 (18.37)	
	5	23 (24.21)	8 (24.24)	15 (30.61)	
Year of diagnosis	≤2021.12	15 (15.79)	5 (15.15)	10 (20.00)	0.574
	>2021.12	68 (50.37)	28 (84.85)	40 (80.00)	

Some numbers are slightly less than n due to partial data uncertainty.

### 3.2 Baseline characteristics of patients after PSM

To Propensity score matching (PSM) with a 1:1 nearest-neighbor algorithm (caliper=0.2) was implemented to mitigate confounding bias. Post-matching analysis demonstrated successful balance in baseline characteristics between immunotherapy (n=20) and non-immunotherapy cohorts (n=20), with standardized mean differences <0.1 for all variables, except M staging (SMD < 0.25). As detailed in **Table 2**, key parameters including age, BMI, gender, marital status, clinical stages, T stage, N stage, M stage, complication, tumor location, and tumor size showed no statistically significant intergroup differences (P>0.05). This methodological rigor ensured comparability for subsequent survival analyses.

**Table 2:** Baseline characteristics after PSM.

Characteristics		Total	Immunotherapy (n=20)	Non-immunotherapy (n=20)	P-value
Age		65.69 ± 9.09	65.00 ± 8.79	66.38 ± 9.56	0.629
BMI		22.09 ± 2.92	21.74 ± 2.61	22.45 ± 3.24	0.441
Tumor size (cm)		4.48 ± 1.65	4.29 ± 1.66	4.67 ± 1.66	0.461
Gender	Male	32 (80.95)	16 (80.00)	16 (80.00)	1.000
	Female	8 (19.05)	4 (20.00)	4 (20.00)	
Marital status	Single	0 (0.00)	0 (0.00)	0 (0.00)	–
	Married	40 (100.00)	20 (100.00)	20 (100.00)	
Clinical Stages	II	2 (5.00)	1 (5.00)	1 (5.00)	0.950
	IIIA	2 (5.00)	1 (5.00)	1 (5.00)	
	IIIB	11 (27.5)	7 (35.00)	4 (20.00)	
	IVA	3 (7.5)	1 (5.00)	2 (10.00)	
	IVB	22 (55.00)	10 (50.00)	12 (60.00)	
T stage	T0-1	3 (7.5)	0 (0.00)	3 (15.00)	0.289
	T2	11 (27.5)	5 (25.00)	6 (30.00)	
	T3	22 (55.5)	13 (65.00)	9 (45.00)	
	T4	4 (1.0)	2 (10.00)	2 (10.00)	
N stage	N0	3 (7.5)	2 (10.00)	1 (5.00)	0.613
	N1	16 (40.0)	6 (30.00)	10 (50.00)	
	N2	12 (30.0)	7 (35.00)	5 (25.00)	
	N3	9 (22.5)	5 (25.00)	4 (20.00)	
M stage	M0	17 (42.5)	10 (50.00)	7 (35.00)	0.533
	M1	23 (57.5)	10 (50.00)	13 (65.00)	
Complication	Yes	27 (67.5)	14 (70.00)	13 (65.00)	0.739
	No	13 (32.5)	6 (30.00)	7 (35.00)	
Tumor location	2	1 (2.5)	0 (0.00)	1 (5.00)	0.449
	3	17 (42.5)	8 (40.00)	9 (45.00)	
	4	12 (30.0)	8 (40.00)	4 (20.00)	
	5	10 (25.0)	4 (20.0)	6 (30.0)	
Year of diagnosis	≤2021.12	8 (20.0)	2 (10.00)	6 (30.00)	0.060
	>2021.12	32 (80.0)	18 (90.00)	14 (70.00)	

PSM, propensity score matching analyses. Complication, including hypertension, diabetes mellitus, chronic obstructive pulmonary disease, pneumonia, cerebral infarction, coronary atherosclerotic heart disease. Tumor location, the segments of esophagus cancer1,2, 3, 4 and 5 represent the cervical, upper thoracic, middle thoracic, lower thoracic and abdominal segments, respectively.

### 3.3 Survival analysis

**Figure 3:**
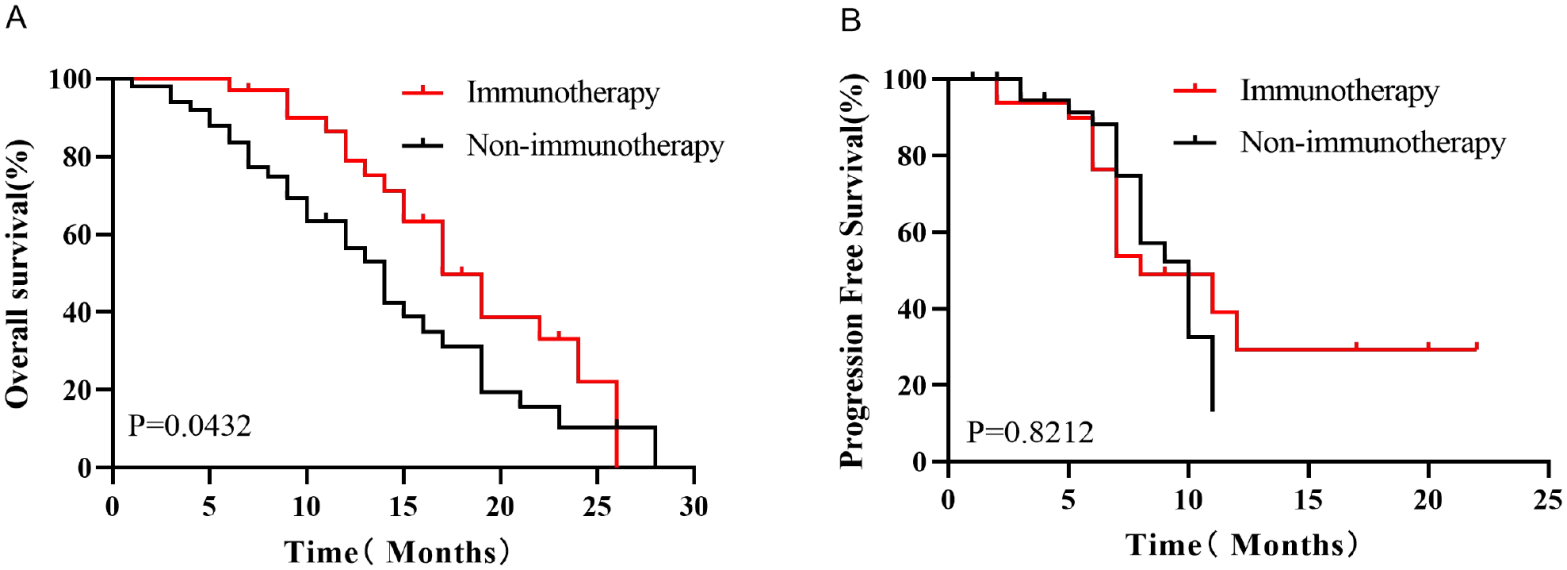
Kaplan-Meier survival analysis of OS **(A)** and PFS **(B)** before PSM.

**Figure 4:**
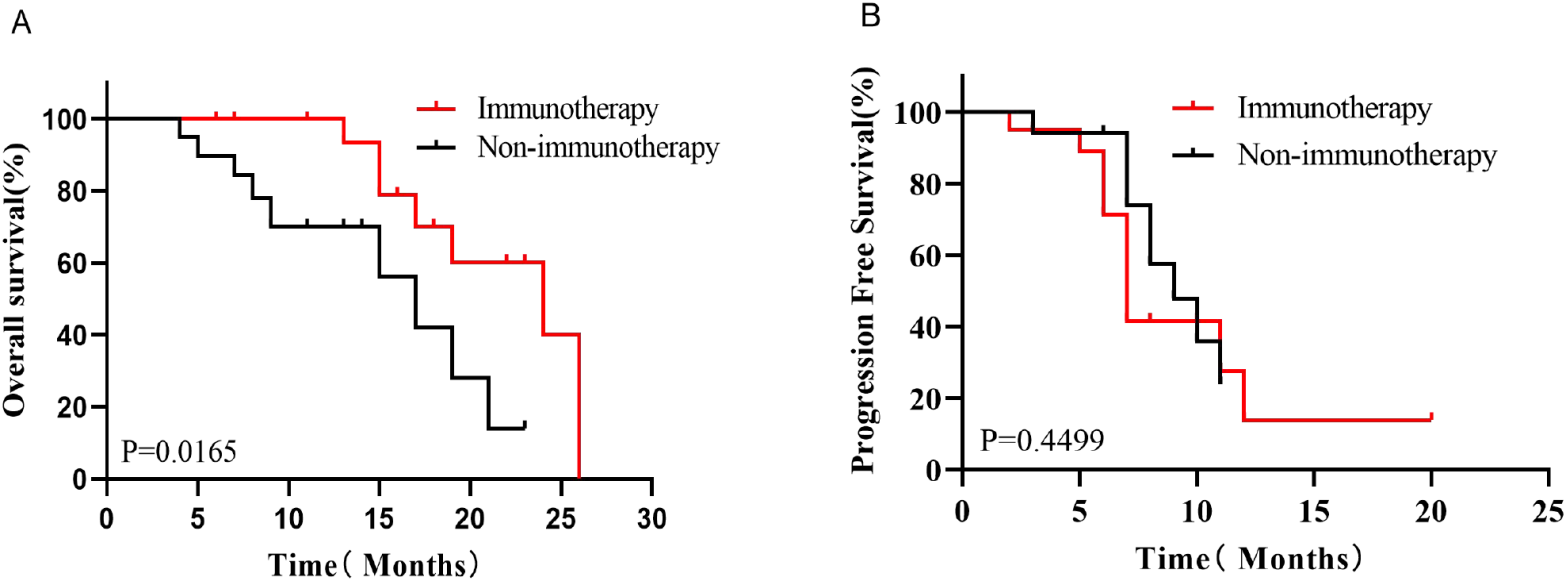
Kaplan-Meier survival analysis of OS **(A)** and PFS **(B)** after PSM.

Kaplan-Meier survival analysis before PSM showed that the median OS was better in the Immunotherapy group than in the Non-immunotherapy group (19 months *vs*. 14 months, P=0.0432, HR=0.5788, 95% CI 0.3324-1.008, **Figure 3A**), while there was no significant difference in the median PFS between the two groups (9 months *vs*. 9 months, P=0.8212, HR=0.9305, 95% CI 0.4681-1.850,**Figure 3B**). After PSM, patients in the Immunotherapy group had a more favorable OS than that in the Non-immunotherapy group (22 months *vs*. 13 months P=0.0165, HR=0.3595, 95% CI 0.1258-1.027,**Figure 4A**). However, no significant difference was observed in median PFS between the Immunotherapy group and the Non-immunotherapy group (9 months *vs*. 7 months P=0.4499, HR=1.3460, 95% CI 0.5723-3.167, **Figure 4B**).

By analyzing the survival rates of patients in the two groups (**Table 3**), the six-month, one-year, and two-year survival rates of patients in the Immunotherapy group were 97.0%, 78.9%, and 22.1%, respectively, while those of the Non-immunotherapy group were 83.7%, 56.5%, and 10.4%, respectively. Patients in the Immunotherapy group had better six-month, one-year, and two-year survival rates than patients in the Non-immunotherapy group.

**Table 3:** Half year, 1-and 2-year survival rate of OS.

Overall Survival Rates (%)	6-month	1-year	2-year
Immunotherapy	97.0%	78.9%	22.1%
Non-immunotherapy	83.7%	56.5%	10.4%

### 3.4 Univariate and multivariate regression analysis of OS and PFS

Univariate and multivariate analyses of overall survival (OS) and progression-free survival (PFS) were conducted for both the Immunotherapy and Non-immunotherapy groups (**Tables 4**, **5**), incorporating all available variables into the initial models. Variables exhibiting a significance level of P < 0.2 were subsequently included in the multivariate Cox analysis, which identified treatment modality as an independent prognostic factor for OS (P=0.039). Conversely, year of diagnosis was identified as an independent prognostic variable for PFS (P=0.031).

**Table 4:** Univariate and multivariate regression analysis of OS.

Factor	Univariate			Multivariate		
	HR^1^	95%CI^1^	P-value	HR^1^	95%CI^1^	P-value
Treatment methods^2^	1.763	0.987, 3.148	**0.055**	2.423	1.045, 5.616	**0.039**
Tumor size(cm)	1.058	0.986, 1.135	**0.119**	1.036	0.956, 1.124	0.388
Age	0.996	0.963, 1.030	0.799	–	–	–
BMI	1.037	0.955, 1.126	0.383	–	–	–
Gender	1.031	0.498, 2.136	0.934	–	–	–
Marital status	0.214	0.050, 0.926	**0.039**	0.152	0.071, 1.344	0.090
Clinical Stages	1.260	1.004, 1.580	**0.046**	1.400	0.815, 2.404	0.223
T stage	1.085	0.738, 1.594	0.679	–	–	–
N stage	1.102	0.778, 1.562	0.584	–	–	–
M stage	1.625	0.859, 3.073	**0.135**	0.455	0.092, 2.244	0.334
Complication	1.076	0.606, 1.910	0.802	–	–	–
Tumor location	0.958	0.715, 1.284	0.775	–	–	–
Year of diagnosis	1.008	0.510, 1.991	0.982	–	–	–

In the multivariate analysis, bold values indicate P < 0.2, while in the univariate analysis table, bold values indicate P < 0.05.

**Table 5:** Univariate and multivariate regression analysis of PFS.

Factor	Univariate			Multivariate		
	HR^1^	95%CI^1^	P-value	HR^1^	95%CI^1^	P-value
Treatment methods^2^	0.991	0.517, 1.901	0.978	–	–	**-**
Tumor size(cm)	1.025	0.903, 1.163	0.705	–	–	–
Age	0.967	0.931, 1.005	**0.086**	0.977	0.924, 1.034	0.420
BMI	1.078	0.970, 1.198	**0.145**	1.101	0.932, 1.301	0.259
Gender	0.969	0.443, 2.119	0.937	–	–	–
Marital status	0.565	0.135, 2.365	0.434			–
Clinical Stages	1.228	0.574, 1.254	**0.102**	1.400	0.815, 2.404	0.897
T stage	0.828	0.738, 1.594	0.373	–	–	–
N stage	1.296	0.895, 1.877	**0.170**	1.022	0.591, 1.765	0.939
M stage	1.706	0.828, 3.513	**0.148**	0.455	0.092, 2.244	0.901
Complication	0.839	0.438, 1.607	0.596	–	–	–
Tumor location	1.075	0.769, 1.504	0.671	–	–	–
Year of diagnosis	0.508	0.254, 1.018	**0.056**	1.042	0.564, 1.924	**0.031**

PFS, progression free survival; OS, overall survival. Complication, including hypertension, diabetes mellitus, chronic obstructive pulmonary disease, pneumonia, cerebral infarction, coronary atherosclerotic heart disease. Tumor location, are esophageal cancer segments. In the multivariate analysis, bold values indicate P < 0.2, while in the univariate analysis table, bold values indicate P < 0.05.

In the regression analyses examining overall survival (OS) and progression-free survival (PFS) in the immunotherapy cohort, key variables including line of therapy, types of immune checkpoint inhibitors (ICIs), and subsequent therapies were evaluated. Factors with a P-value <0.2 in univariate analysis were further included in multivariate models (**Tables 6**, **7**).

**Table 6:** Univariate and multivariate regression analysis of OS (immunotherapy group).

Factor	Univariate			Multivariate		
	HR^1^	95%CI^1^	P-value	HR^1^	95%CI^1^	P-value
Line of Therapy	0.251	0.330, 1.921	**0.183**	0.225	0.029, 1.726	0.151
Types of ICIs	0.391	0.500, 3.067	0.342	–	–	–
Subsequent Treatment	0.555	0.308, 0.998	**0.049**	0.641	0.312, 0.925	**0.041**

In the multivariate analysis, bold values indicate P < 0.2, while in the univariate analysis table, bold values indicate P < 0.05.

**Table 7:** Univariate and multivariate regression analysis of PFS (immunotherapy group).

Factor	Univariate			Multivariate		
	HR^1^	95%CI^1^	P-value	HR^1^	95%CI^1^	P-value
Line of Therapy	0.683	0.860, 5.397	0.717	–	–	–
Types of ICIs	0.265	0.350, 2.030	**0.200**	0.477	0.570, 3.964	0.493
Subsequent Treatment	0.582	0.330, 1.024	**0.061**	0.624	0.343, 1.137	0.123

PFS, progression free survival; OS, overall survival. Line of Therapy: Including first-line therapy, second-line therapy, third-line therapy, and fourth-line therapy; Types of ICIs (Immune Checkpoint Inhibitors): Camrelizumab, Serplulimab, Toripalimab, Sintilimab, Pembrolizumab, Tislelizumab, Adebrelimab, and Envafolimab. Of these, Adebrelimab and Envafolimab are PD-L1 inhibitors, while the remaining agents are classified as PD-1 inhibitors; Subsequent Treatment: Continued antitumor therapy (excluding immunotherapy), symptomatic treatment, death during immunotherapy, stable disease status post-immunotherapy (with no further treatment administered). In the multivariate analysis, bold values indicate P < 0.2, while in the univariate analysis table, bold values indicate P < 0.05.

For OS, multivariate analysis identified subsequent therapies as an independent prognostic factor (P = 0.041). In contrast, none of the evaluated variables—line of therapy, types of ICIs, or subsequent therapies—demonstrated statistically significant associations with PFS in multivariate analysis (all P > 0.05). These findings suggest that while subsequent treatment modalities significantly influence long-term survival outcomes in patients receiving immunotherapy, they do not appear to impact short-term disease progression.

## 4 Discussion

This dual-center retrospective cohort study investigated immunotherapy efficacy in small cell carcinoma of the esophagus (SCCE) through parallel data collection at two tertiary care institutions. SCCE represents a rare, highly aggressive neuroendocrine malignancy exhibiting rapid disease progression, early metastatic dissemination(predominantly to liver [40%], lungs [35%], and lymph nodes [60%]), and dismal survival outcomes (2, 13, 14). Current evidence indicates 5-year survival rates of 10.2% for limited-stage disease versus <2% for extensive-stage presentations (1). The 5-year survival rate for patients diagnosed with extensive stage is virtually negligible (4, 5). Previous studies have explored various treatment modalities for SCCE. Meng et al. found that radiotherapy had a better OS than postoperative chemotherapy in limited stage SCCE (15). Chen et al. concluded that surgical resection is the mainstay of treatment for stage I or II primary small cell carcinoma of the esophagus(PSCCE) and that chemotherapy does not further improve survival (16). Another study suggested that preoperative chemotherapy combined with surgery improves overall survival in patients with limited-stage SCCE compared with upfront surgery (17). And according to some studies that have put forward the idea that stage III or IV patients should receive chemotherapy and radiation (18). Another study by Li et al. concluded that chemotherapy and radiotherapy improved OS and cancer-specific survival (CSS) in SCCE patients (19). And OS was better in the chemoradiotherapy (CRT) group (18 months) compared to radiation alone (10 months) (20). However, studies on immunotherapy for SCCE are scarce, and there are no large clinical data investigating the role of immunotherapy in SCCE, except for case reports.

Our analysis demonstrated significantly prolonged OS in the immunotherapy cohort compared to non-immunotherapy controls. This survival benefit persisted on multivariable Cox regression where immunotherapy emerged as an independent prognostic determinant(P=0.039). The observed male predominance (72% *vs* 28% female) aligns with prior epidemiological reports (1, 21, 22), potentially reflecting differential smoking patterns or hormonal influences. Notably, while clinical stage and M-category demonstrated prognostic significance in previous series (21, 23), these associations were not replicated in our cohort - a discrepancy potentially attributable to methodological variations in staging protocols or population heterogeneity. In addition, our study showed that patients in the immunotherapy group had longer OS than those in the non-immunotherapy group, suggesting that immunotherapy may improve the prognosis of patients with SCCE, but there was no significant difference in PFS between the two groups, and we analyzed the reasons for this phenomenon. First, this phenomenon may be attributed to the delayed treatment effect characteristic of immunotherapy. Immune activation requires a certain period to manifest clinically, immune checkpoint inhibitors (e.g., PD-1/PD-L1 inhibitors) exert anti-tumor effects by activating T cells, a process that may take weeks to months. During this latency phase, tumors might exhibit no significant reduction or even transient enlargement due to pseudoprogression (caused by immune cell infiltration or inflammatory responses), thereby obscuring short-term PFS improvement. Second, the establishment of long-lasting anti-tumor immune memory could contribute to OS prolongation. Once fully activated, the immune system may develop persistent immunological memory, which reduces late-phase recurrence or mortality during extended follow-up. However, such delayed protective effects predominantly enhance OS rather than early-phase disease control metrics like PFS. Finally, the distinct biological implications of OS and PFS endpoints should be considered. While PFS primarily reflects the speed of tumor control (e.g., direct cytoreduction), OS encompasses comprehensive survival benefits, including delayed recurrence, effective management of secondary malignancies, and enhanced immune surveillance. Immunotherapy may extend survival through mechanisms beyond immediate tumor killing (e.g., remodeling the tumor microenvironment or sustaining systemic anti-tumor immunity), which aligns with observed OS advantages despite nonsignificant PFS differences.

SCCE and SCLC exhibit significant similarities in histopathology, biological behavior, and molecular characteristics, including neuroendocrine differentiation, highly aggressive behavior, and early metastatic potential. Consequently, SCCE treatment protocols frequently align with SCLC guidelines. For instance, the EP regimen (Etoposide plus Cisplatin), a cornerstone chemotherapy for SCLC, has been adopted for systemic therapy in SCCE. Notably, among the 83 enrolled SCCE patients in our study, 51 (61.4%) received either the EP regimen or EP combined with immunotherapy, further validating this clinical practice. A study by Chen et al. (16) also suggested that clinical decision-making in patients with SCCE is dependent on treatment experience in SCLC. In their study, the combination of Etoposide and platinum-based chemotherapy was the most widely used first-line regimen, which is similar to the data in our study. Several studies (20, 24) have also concluded that SCEC and SCLC have similar pathological features and biological behaviors, and therefore it is reasonable to use the same treatment regimen for both cancers.

Despite the current treatment of SCCE refers to the treatment of esophageal gland/squamous cell carcinoma (EAC/ESCC) and small cell lung cancer (SCLC), the prognosis of SCCE patients is significantly worse than that of EAC/ESCC and SCLC (25, 26). Typically, patients with SCCE have a median survival of 8–13 months, with a 2-year survival rate of approximately 20%. While chemotherapy is initially effective, most patients experience rapid relapse and limited responses to subsequent treatments. The necessity for more effective and precise treatment strategies for SCCE is paramount in clinical settings (3). Immunotherapy has emerged as a significant breakthrough in the field of cancer treatment, thereby profoundly impacting tumor treatment strategies (27). ICIs have become integral to contemporary therapeutic strategies, representing a cornerstone advancement among immunotherapeutic approaches (28). ATTRACTION-3 (29) is a phase III trial comparing Nivolumab (anti-PD-1) *vs* chemotherapy in advanced ESCC after first-line therapy. Nivolumab demonstrated superior OS (median 10.9 *vs* 8.5 months; HR=0.79) and became the first PD-1 inhibitor approved in Japan (2020) for chemotherapy-refractory ESCC. KEYNOTE-181 (30) established Pembrolizumab’s efficacy in PD-L1 CPS≥10 esophageal cancer (including ESCC and adenocarcinoma) as second-line therapy. Median OS improved to 9.3 *vs* 6.7 months (HR=0.69) in CPS≥10 patients, leading to FDA approval (2019) for PD-L1-positive ESCC. In the CheckMate -648 (31) trial, the O+Y group demonstrated a significant overall survival (OS) benefit compared to the chemotherapy-alone group regardless of PD-L1 expression levels. Furthermore, the O+Y group exhibited a prolonged median duration of response (DoR), reaching 11.1 months in the overall population — 1.5 times longer than that of the chemotherapy-alone group (7.1 months). Notably, this improvement was even more pronounced in patients with tumor PD-L1 expression ≥1%, where the median DoR in the O+Y group more than doubled compared to the chemotherapy-alone group (11.8 months *vs*. 5.7 months).These trials collectively validated PD-1/PD-L1 inhibitors in ESCC across treatment lines and supported biomarker-driven approvals, reshaping therapeutic paradigms.

From a mechanistic perspective, immune checkpoints are defined as molecules belonging to co-inhibitory signaling pathways that facilitate the maintenance of immune tolerance. However, these molecules are frequently utilized by cancer cells to evade immune surveillance (32). The objective of ICIs is to reinvigorate immune responses that target tumors, whilst concomitantly facilitating the immune-mediated eradication of neoplastic cells by disrupting the signaling pathways that act as regulatory mechanisms for T-cell activity. These treatments have elicited notable outcomes in a range of oncological settings, and there has been an observed augmentation in the scope of indications for which this approach is employed (22, 33, 34). Current therapeutic paradigms for ESCC demonstrate that immune checkpoint inhibitor (ICI)-chemotherapy combinations now constitute first-line standard care for advanced-stage disease (35), particularly in PD-L1-positive populations as evidenced by survival improvements in pivotal trials (36). While SCCE-specific ICI data remain scarce, emerging case series support synergistic efficacy of chemoimmunotherapy in locally advanced/metastatic SCCE (37, 38), corroborating our survival analyses. Notably, Wu et al. recently documented the first successful neoadjuvant chemoimmunotherapy (NACI) protocol for resectable SCCE, achieving major pathological response through PD-1 blockade combined with platinum-doublet chemotherapy (39). This paradigm-shifting report suggests NACI merits prospective validation as a potential curative-intent strategy for localized SCCE.

Biomarker-driven approaches appear critical, as SCLC studies identify T-cell-inflamed microenvironments (dual PD-L1+/CD8+ T-cell infiltration) as predictors of ICI responsiveness (18). Translational SCCE analyses confirm this PD-L1/CD8+ co-expression pattern correlates with improved survival (40), suggesting analogous patient selection criteria may optimize SCCE immunotherapy. Therapeutic insights from small cell lung cancer (SCLC) mechanistically inform SCCE management, given their shared molecular pathogenesis involving SOX2 overexpression and Rb1/p53 pathway inactivation (41). These biological parallels further rationalize adapting SCLC treatment algorithms, where chemoimmunotherapy combinations now represent the evidence-based standard for extensive-stage disease (42), to SCCE clinical practice. In a similar vein, a combination of chemotherapy and immunotherapy has been widely accepted as the primary treatment for advanced esophageal squamous cell carcinoma(ESCC) (38). This findings indicate that combination immunotherapy holds potential clinical value for SCCE patients, warranting further investigation. To some extent, this study contributes to addressing the existing knowledge gap in immunotherapy for SCCE.

Zhu et al. (25) compared the efficacy of two chemotherapy regimens commonly used in current SCCE treatment, namely Etoposide plus Cisplatin (EP) and Irinotecan plus Cisplatin (IP), however, no significant differences were observed in OS and disease-free survival(DFS). Additionally, while these studies suggested potential survival benefits of immunotherapy in SCCE, no conclusive data are available to confirm this this observation. Our study also analyzed the half-year, one-year and two-year survival rates of the immunotherapy group and the non-immunotherapy group, survival analysis revealed significantly higher 6-month and 1-year survival rates in the immunotherapy cohort compared to the control group, though no significant difference was observed at 2-year follow-up.

However, some evidence suggests that achieving the desired results in SCCE with chemotherapy combined with immunotherapy may be challenging. A study noted that the tumor microenvironment (TME) of PSCCE is characterized by inadequate T-cell infiltration (8). However, the most widely used targets of ICIs are cytotoxic T-lymphocyte-associated molecule-4 (CTLA-4), programmed cell death receptor-1 (PD-1), and programmed cell death ligand-1 (PD-L1), all of which act by blocking the T-cell inhibitory pathway. Nevertheless, primary esophageal small cell carcinoma (PSCCE) demonstrates limited T-cell infiltration within the tumor microenvironment, potentially compromising the efficacy ICIs. Further studies are warranted to elucidate the therapeutic potential of ICIs in SCCE and their impact on long-term prognosis.

Emerging clinical observations underscore the superiority of multimodal therapy over monotherapy in SCCE management. A representative case documented sustained near-complete remission (CR) and survival exceeding 19 months in extensive-stage SCCE following chemoimmunotherapy (43). To systematically evaluate this paradigm, we conducted a multi-institutional retrospective analysis of treatment-naive SCCE patients (n=121) from two tertiary centers (Provincial Hospital and Cancer Hospital of Shandong First Medical University, 2019–2024). After applying PSM to minimize confounders, our cohort demonstrated significantly prolonged OS in the immunotherapy group versus non-immunotherapy group (P=0.0165, HR=0.3595, 95% CI 0.1258-1.027). This study provides evidence supporting chemoimmunotherapy as a clinically meaningful intervention in advanced SCCE, though validation through prospective trials remains imperative.

This study represents the first systematic investigation of immunotherapy in SCCE, offering valuable insights into its potential benefits. By employing PSM, we minimized confounding factors and ensured robust comparisons. It is essential to recognize the constraints of this study, even though it has provided helpful information. 1. Limited Sample Size: The retrospective nature of this study inherently restricted the sample size, which may reduce statistical power and limit the generalizability of our findings. A smaller cohort increases the risk of potentially obscuring clinically meaningful differences in outcomes. Furthermore, although we listed all the ICIs used in the study in detail, we did not analyze the efficacy of the different ICIs because of the small sample size and the number of patients used for each ICI was too small. 2. Focus on OS and PFS Alone: Due to the limited data that could be collected, our analysis was confined to overall survival (OS) and progression-free survival (PFS) as primary endpoints. While these metrics are clinically relevant, they do not capture other critical aspects of treatment efficacy, such as quality of life, symptomatic burden, or treatment-related toxicity. Future studies should incorporate patient-reported outcomes and granular toxicity data to provide a more holistic evaluation. 3. Residual Bias Despite PSM: Although propensity score matching (PSM) was employed to mitigate baseline imbalances between cohorts, this method only balances known confounders in the data. Unknowable variables (e.g., genetic profiles, socioeconomic status, or undiagnosed comorbidities) may persist as sources of residual bias, potentially distorting the observed associations. Moreover, PSM reduced the effective sample size, further amplifying uncertainties in the estimates. More samples and data are needed in the future to further validate the findings of the study.

## 5 Conclusion

This study revealed that combination immunotherapy regimens significantly improved OS in patients with SCCE compared to conventional chemotherapy-based therapies, although no statistically significant differences in PFS were observed. These findings underscore the therapeutic potential of immune checkpoint inhibitor-based strategies for SCCE management, warranting further validation through prospective multicenter randomized controlled trials.

## Data Availability

The raw data supporting the conclusions of this article will be made available by the authors, without undue reservation.
